# Metformin as a Metabolic Reprogramming Interface in Host–Pathogen and Bone Microenvironment Crosstalk: A Dual-Target Strategy Against Antimicrobial Resistance and Osteoporotic Bone Loss

**DOI:** 10.3390/antibiotics15060583

**Published:** 2026-06-08

**Authors:** Shakta Mani Satyam, Ebrahim Safaii, Ilmia Shameer, Rashmi Kumari, Sainath Prabhakar, Mohamed Talat Zaky Mahmoud Eltrabishi, Mohamed El-Tanani, Abdul Rehman, Mohamed Tarek Mohamed Wageh Mohamed Abdelfattah

**Affiliations:** 1 Department of Pharmacology, RAK College of Medical Sciences, RAK Medical and Health Sciences University, Ras Al Khaimah 11172, United Arab Emirates; 2 A. J. Hospital and Research Centre, A. J. Institute of Hospital Management, Mangalore 575004, India; 3 Department of Perfusion Technology, Manipal College of Health Professions, Manipal Academy of Higher Education, Manipal 576104, India; 4 RAK College of Pharmacy, RAK Medical and Health Sciences University, Ras Al Khaimah 11172, United Arab Emirates; 5 Department of Pathological Sciences, College of Medicine, Ajman University, Ajman P.O. Box 346, United Arab Emirates

**Keywords:** metformin, antimicrobial resistance, osteoimmunology, bone remodeling, AMPK signaling, mitochondrial dysfunction, biofilm infection, metabolic reprogramming

## Abstract

Metabolic dysregulation is increasingly recognized as a central feature linking chronic infection, immune dysfunction, and skeletal deterioration; however, these processes are most often investigated in isolation, limiting the development of integrative mechanistic frameworks. In this review, we propose the Metabolic Reprogramming Interface Model (MRIM) as a systems-level, hypothesis-generating construct that conceptualizes metabolism as a shared regulatory axis bridging host–pathogen interactions and bone microenvironment remodeling. Importantly, MRIM is not presented as a unified or experimentally validated disease model, but rather as a structured framework designed to organize and critically evaluate emerging multidisciplinary evidence. At the molecular level, metformin, a widely used metabolic modulator, has been shown to influence mitochondrial bioenergetics, AMP-activated protein kinase (AMPK) signaling, redox balance, and autophagic pathways, all of which are independently implicated in microbial persistence, immune cell function, and skeletal homeostasis. Within MRIM, these observations are integrated to hypothesize that metabolic perturbation may coordinately influence infection dynamics, inflammatory responses, and bone turnover. Nevertheless, most of the supporting evidence remains indirect, arising from in vitro studies, animal models, and observational clinical datasets, thereby limiting causal inference. To address this, the framework explicitly distinguishes between experimentally validated mechanisms, context-dependent biological interactions, and higher-order theoretical integrations. While preliminary findings suggest that metformin may modulate microbial fitness, attenuate excessive inflammation, and influence bone remodeling, these effects appear to be highly context-dependent and have not yet been substantiated in adequately powered prospective clinical trials evaluating combined infectious and skeletal outcomes. This review therefore provides a critical synthesis of current knowledge, highlights key mechanistic and translational uncertainties, and outlines testable hypotheses for future investigation, positioning MRIM as a conceptual scaffold to guide interdisciplinary research rather than a definitive explanatory model.

## 1. Introduction

Antimicrobial resistance (AMR) and osteoporosis pose significant global public health threats. AMR has been projected to cause up to 10 million deaths annually worldwide by 2050 [1,2]. The main drivers of AMR include the overuse and misuse of antimicrobials, the failure of new antimicrobial compounds entering clinical development and the rapid evolution of microbes to become resistant to current and newly introduced antimicrobial agents. Large-scale global burden analyses have estimated that bacterial AMR was associated with approximately 4.71 million deaths in 2021, including 1.14 million deaths directly attributable to resistance, with projections suggesting a rise to nearly 8.22 million associated deaths annually by 2050, particularly affecting older populations and regions with limited healthcare access [1]. Simultaneously, osteoporosis affects hundreds of millions of individuals globally, particularly postmenopausal women and the aging population, leading to increased fracture risk, morbidity, and healthcare costs [3]. Epidemiological assessments from global disease burden datasets further indicate that low bone mineral density (BMD) accounted for over 219,000 deaths and 7.76 million disability-adjusted life years in postmenopausal women in 2021, with absolute mortality more than doubling since 1990, largely driven by population aging despite modest declines in age-standardized rates [4,5]. While these two disease entities are conventionally studied in isolation, one as an infectious disease challenge and the other as a degenerative skeletal disorder, emerging evidence suggests a profound biological convergence between microbial persistence, chronic inflammation, and skeletal deterioration.

In this context, metformin has emerged as a pharmacological agent of interest not only for its glucose-lowering properties but for its capacity to directly modulate cellular bioenergetics at the mitochondrial level. Mechanistically, metformin partially inhibits complex I of the mitochondrial electron transport chain, resulting in reduced oxidative phosphorylation (OXPHOS), decreased adenosine triphosphate (ATP) generation, and an increased adenosine monophosphate (AMP)/adenosine triphosphate (ATP) ratio [6]. This bioenergetic perturbation activates AMP-activated protein kinase (AMPK) and induces a compensatory metabolic shift toward glycolysis [7]. Importantly, this OXPHOS-to-glycolysis transition represents a primary upstream event, from which downstream effects on inflammation, immune function, and cellular differentiation emerge [8,9]. Accordingly, this review proposes the Metabolic Reprogramming Interface Model (MRIM) as a conceptual framework that may help explain how metabolic adaptation and resource utilization across host, pathogen, and skeletal compartments could contribute to disease progression under selected pathological conditions.

A growing body of research now supports the concept that infection-driven inflammation contributes significantly to bone loss [10,11,12]. Chronic infections caused by pathogenic bacteria and fungi such as *Candida albicans*, *Staphylococcus aureus*, and Gram-negative bacteria can establish chronic inflammatory microenvironments that disrupt normal bone homeostasis [13,14,15]. In their biofilm form, these pathogens not only become more persistent through metabolic changes but can also develop strategies to evade the host immune system [10,16,17]. The resulting low-grade inflammation may persist for long periods, continuously producing pro-inflammatory cytokines such as tumor necrosis factor-alpha (TNF-α), interleukin-1 beta (IL-1β), and interleukin-6 (IL-6), which induce osteoclast formation and bone resorption [18,19]. In addition to immune modulation, another mechanism of bone destruction induced by infection is oxidative stress. During infection, reactive oxygen species (ROS) are produced by host cells in large amounts, which can cause oxidative damage, disrupt normal function of osteoblasts, induce differentiation of host cells into osteoclasts for bone resorption, and contribute to bone destruction [20]. Chronic osteomyelitis, periodontitis, and orthopedic device-related infection (ODRI) are three infectious diseases that cause destruction of bone tissue in the context of chronic microbial colonization of infected tissue. Infected bone tissue can also promote microbial colonization and persistence through its changed architecture and reduced vascularity and is likely to destroy osteoporotic bone in a mutually detrimental interaction [21].

Emerging evidence from diverse experimental systems suggests that oxidative stress regulation, mitochondrial function, and immunometabolic signaling represent common biological processes linking chronic inflammation, tissue degeneration, and host adaptation. Although these observations originate from heterogeneous disease models, they collectively support further investigation of metabolism as a potential integrative framework for understanding complex disease interactions [22,23,24]. While microbial-driven processes and host–pathogen interactions play critical roles in bone infection and destruction, treatments for these conditions are quite distinct from those for treating infection in general and quite different from those for osteoporosis. Antimicrobial therapies are used to treat infections, but they have well recognized limitations including the emergence of resistance, poor penetration into biofilms, and failure to treat chronic infections. In contrast, treatments for osteoporosis have focused on modulating bone remodeling with bisphosphates (e.g., ibandronate, alendronate, zoledronate) and more recently with denosumab and anabolic agents (e.g., teriparatide). There is no current treatment that targets both microbial survival and bone destruction. The need for a more integrated approach to the treatment of bone infection and bone destruction has become increasingly apparent.

Metabolic regulation connects infection biology and bone homeostasis through cellular energy metabolism, including the mitochondrial function and nutrient-sensing pathways that are essential for host cell functions. To survive and cause diseases, pathogens exploit these host metabolic pathways for their own benefit. In host cells, AMPK plays a crucial role in maintaining energy balance and stress resistance. As a multifunctional energy-sensing kinase, AMPK regulates mitochondrial biogenesis and function, autophagy, redox homeostasis, and inflammation. Activation of AMPK or its upstream kinases by physical exercise, muscle contraction, and environmental stresses, as well as by various pharmacological and nutraceutical agents, has been associated with broad protective effects in metabolic diseases, chronic inflammatory disorders, and degenerative diseases [25,26]. Enhanced nuclear factor erythroid 2-related factor 2 (NRF2)-mediated antioxidant defense and improved mitochondrial function have been shown to exert protective effects across diverse disease models, including conditions associated with antimicrobial resistance [27,28,29]. The findings present a systems-level perspective on a single, unified control network that enables host cells to utilize metabolic pathways to defend against chronic diseases.

Metformin, a first-line therapeutic agent for type 2 diabetes mellitus, has recently emerged as a pleiotropic metabolic modulator with far-reaching biological effects beyond glucose regulation. Its primary mechanism involves inhibition of mitochondrial complex-I, affecting energy production in cells and activating the energy-sensing kinase AMPK, resulting in metabolic effects on oxidative stress, autophagy and inflammation [30,31,32]. These same pathways are utilized by microbes to survive and are crucial in bone remodeling [33,34].

In addition to metformin’s host-directed actions to control glycemia, reduce inflammation, and enhance antimicrobial functions of macrophages through metabolic reprogramming, there are studies suggesting that metformin has direct antimicrobial effects. There is evidence that metformin has direct fungicidal activity against *Candida albicans*. Recently, Zhao et al. (2025) reported that metformin inhibits *Candida albicans* biofilm formation through disruption of mitochondrial function that leads to decreased membrane potential and increased ROS production [35]. In addition to inhibition of biofilm formation, metformin suppressed fungal morphogenesis. Most notably, metformin inhibited yeast-to-hyphae transition, a critical virulence attribute for many pathogenic fungi [35,36].

Metformin has been explored as a putative osteoprotector in preclinical and clinical studies [37]. Metformin enhances osteogenic differentiation of mesenchymal stem cells (MSCs) and osteoblast precursor cells through activation of AMPK [38]. In addition, metformin promotes mitochondrial biogenesis via PGC-1α (peroxisome proliferator-activated receptor gamma coactivator 1) activation to support energy generation for bone formation [39]. Metformin attenuates bone resorption by inhibiting RANKL-mediated activation of NF-κB signaling in osteoclast precursors and by upregulating osteoprotegerin (OPG), a decoy receptor that sequesters RANKL and disrupts RANKL–RANK signaling required for osteoclastogenesis [40,41]. The antioxidant effect of metformin further ameliorates bone loss induced by oxidative stress [42].

Previous studies have primarily focused on metformin’s antimicrobial and osteoprotective effects. However, the broader spectrum of its biological actions requires conceptual integration within a mechanistic framework that incorporates the multiple signaling and metabolic pathways influenced by metformin. Most studies to date have focused on either the antimicrobial effects of metformin, or its effects on bone, without appreciating the full metabolic network of effects that metformin implements to decrease glycemia and modulate a wide range of processes including mitochondrial function, ROS production, autophagy and gene expression of inflammatory genes. Despite these advances, a critical conceptual gap remains in integrating the antimicrobial and osteoprotective roles of metformin into a unified mechanistic framework.

To enhance conceptual clarity and avoid potential misinterpretation, this review is structured with a primary emphasis on metformin as a modulator of cellular energy metabolism within the proposed MRIM framework, rather than as a direct antimicrobial or anti-osteoporotic agent. Antimicrobial resistance and infection-associated bone loss are therefore considered as clinically relevant contexts in which metabolic reprogramming may play a contributory, but not exclusive, role. This distinction is essential, as it positions MRIM not as a disease-specific model, but as a systems-level hypothesis that integrates metabolic regulation with immune responses and tissue remodeling processes. By framing these interactions within a unified metabolic perspective, the review seeks to bridge traditionally separate domains while maintaining clear boundaries between established evidence and conceptual extrapolation.

## 2. Literature Search Strategy

This narrative review employed a structured, transparent, and reproducible literature search strategy designed to integrate mechanistic, preclinical, translational, and clinical evidence related to the emerging role of metformin as a metabolic reprogramming interface across host–pathogen interactions and bone microenvironment regulation. Given the interdisciplinary and hypothesis-generating nature of the proposed Metabolic Reprogramming Interface Model (MRIM), a narrative synthesis approach was selected to enable integration of diverse mechanistic domains spanning immunometabolism, microbial adaptation, mitochondrial signaling, inflammatory regulation, osteoimmunology, and skeletal remodeling. A comprehensive literature search was conducted using the PubMed search engine and Scopus database to identify relevant studies published between January 2010 and March 2026. The search strategy incorporated both Medical Subject Headings (MeSH) and free-text terms combined using Boolean operators (AND, OR). Representative search combinations included: (“metformin” AND “antimicrobial resistance”), (“metformin” AND “*Candida albicans* biofilm”), (“metformin” AND “*Pseudomonas aeruginosa*”), (“metformin” AND “*Mycobacterium tuberculosis*”), (“metformin” AND “*Staphylococcus aureus* biofilm”), (“metformin” AND “mitochondrial dysfunction”), (“metformin” AND “AMPK”), (“metformin” AND “autophagy”), (“metformin” AND “osteoporosis”), (“metformin” AND “RANKL signaling”), (“metformin” AND “infection-induced bone loss”), (“metformin” AND “osteomyelitis”), and (“metformin” AND “host–pathogen metabolism”). Additional supplementary searches incorporated terms related to microbial biofilm metabolism, mitochondrial complex-I modulation, oxidative stress signaling, macrophage polarization, osteoclastogenesis, osteoblast differentiation, redox biology, and immunometabolic regulation. Studies were considered eligible if they provided mechanistic, experimental, preclinical, translational, or clinical evidence relevant to metformin-mediated modulation of microbial systems, inflammatory signaling, mitochondrial bioenergetics, host immune responses, skeletal remodeling, or bone–infection crosstalk. Priority was given to peer-reviewed original research articles, mechanistic investigations, experimental infection models, translational studies, and clinically relevant observational research. Selected review articles were included when they provided substantial conceptual synthesis or foundational mechanistic context directly relevant to the MRIM framework. Exclusion criteria included non-peer-reviewed literature, editorials, opinion-based commentary lacking primary evidence, duplicate publications, incomplete datasets, conference abstracts without full reports, studies lacking mechanistic or translational relevance, and articles unrelated to immunometabolic or skeletal–pathogen interactions. To enhance comprehensiveness and minimize omission of influential studies, manual reference list screening and forward citation chaining were additionally performed for all major mechanistic and translational articles identified during the primary search process. Only English-language peer-reviewed publications were included. Titles and abstracts were initially screened for conceptual relevance, mechanistic depth, and translational applicability, followed by detailed full-text evaluation of selected studies. Duplicate records identified across databases were removed manually. As this manuscript was designed as a narrative conceptual review rather than a formal systematic review or meta-analysis, PRISMA-based quantitative selection methodology was not applied. Nevertheless, methodological rigor and reproducibility were prioritized through structured screening and evidence stratification procedures. The initial search process identified more than 450 potentially relevant publications, of which approximately 180 underwent full-text assessment based on scientific quality, mechanistic relevance, and translational significance. Ultimately, 117 references were selected for inclusion based on their contribution to the conceptual development, mechanistic support, and translational interpretation of the MRIM framework. This structured methodology enabled a rigorous and conceptually integrated synthesis of the emerging evidence supporting metformin as a potential immunometabolic regulator beyond its traditional glucose-lowering role. It also allowed critical evaluation of current limitations, translational uncertainties, and future research directions.

## 3. Mechanistic Basis of Antimicrobial Action of Metformin

### 3.1. Metabolic Reprogramming as an Antimicrobial Paradigm

Unlike traditional antimicrobials that target specific metabolic pathways by inactivating enzymes, metformin has been reported to influence host and microbial metabolic pathways through modulation of cellular bioenergetics and stress-response mechanisms, although the magnitude and biological relevance of these effects appear to vary considerably across experimental systems. The diverse effects of metformin on microbes include inhibition of mitochondrial energy production, uncoupling of oxidative phosphorylation and increased ROS production that can alter membrane homeostasis and stress response [43,44]. Moreover, the effects of metformin on microbes can be predicted by analyzing the homeostasis of a microorganism in stress conditions [45]. Importantly, metformin has been proposed as a candidate for investigation in persistent infections and biofilm-associated diseases; however, evidence supporting such applications remains predominantly preclinical [46].

### 3.2. Mitochondrial Complex-I Inhibition and Bioenergetic Collapse

Metformin’s antimicrobial effects are mediated by inhibition of mitochondrial complex I, leading to excessive electron leakage within the electron transport chain, reduced mitochondrial membrane potential, an increased AMP/ATP ratio indicative of cellular energy depletion, and consequent loss of microbial cell structural integrity [35,47]. In eukaryotic pathogens, such as fungi, mitochondrial respiration serves not only as a source of energy but also as a critical regulatory point where metabolism is linked to morphogenesis, virulence and adaptation to environmental cues [48]. By inhibiting mitochondrial complex- I, metformin prevents electron transport thereby abolishing the formation of the electrochemical proton gradient across the inner membrane and resulting in failure to produce ATP. Consequently, energy-dependent functions in microbial cells including ion transport, membrane homeostasis and biosynthetic processes such as glycolysis, chitin synthesis and actin organization are impaired. As a result of decreased energy, cells lose oxidative sensing nucleic acids structural integrity and are unable to adapt to changing environmental conditions resulting in cessation of growth and cell collapse [49,50,51]. Impairment of fungal bioenergetics can have particularly severe consequences for fungal pathogens that rely on mitochondrial respiration for dealing with stress or nutrient-poor environments and thus constitute less than ideal targets for pharmacological therapy due to their heightened sensitivity to impairment of oxidative phosphorylation.

### 3.3. Oxidative Stress Amplification and Macromolecular Damage

The effects of metformin on mitochondria function induce oxidative stress via increasing electron leakage from impaired respiratory complexes [51]. The generated ROS such as superoxide and hydrogen peroxide can cause severe damage to pathogens that have limited oxidative buffering capacities different from those found in mammalian cells [52]. Even under normal conditions, mammalian cells possess complex mechanisms of antioxidant defenses, and the increased oxidative stress generated by pharmacologically inducing metabolic stress does not cause excessive damage to most cell components. However, in many pathogenic microorganisms, ROS can cause oxidative damage to crucial biomolecules including lipids, resulting in lipid peroxidation that can alter membrane fluidity and increase membrane permeability, leading to membrane instability and perturbation of ion homeostasis. Recent studies have found that oxidative damage to proteins can inactivate enzymes and denature essential proteins, while oxidative damage to oxidative sensing nucleic acids can generate irreparable replication and transcription errors that reduce the viability of the pathogen [53,54,55]. The sum of these injuries leads to loss of homeostasis in microbial cells and their subsequent death.

### 3.4. Autophagy Modulation in Planktonic and Biofilm States

Autophagy is an essential process for intracellular degradation and recycling that is conserved in almost all domains of life. Autophagy can have opposite effects on microbial survival [56,57]. Metformin induces autophagy in many microbial species but its effects on survival duration are context dependent. In growing ‘planktonic’ cells, metformin initially increases survival by inducing autophagy to permit nutrient recovery from damaged organelles and broken-down macromolecules [58]. However, persistent mitochondrial damage and long-term energy deficits caused by metformin subsequently convert this adaptive process to a maladaptive process leading to self-digestion and cell death in contrast to its life-saving effects in nutrient-poor biofilms. In these communities, autophagy is crucial for long-term survival under nutrient-poor conditions and hypoxic microenvironments where cells must maintain tight control over autophagic flux [59]. Using structured populations to dissect interactions between a host and its microbiota, metformin has been reported to inhibit host autophagy which can disrupt balanced host–microorganism interactions [60]. The effects of metformin on microbial metabolism reflect a distinct mechanism of action that targets autophagy, crucial for long-term persistence in various microbial states, thereby offering improved therapeutic efficacy for both acute and chronic diseases while reducing the potential for selection of resistant microorganisms.

### 3.5. Virulence Suppression and Biofilm Disruption

Metformin has been found to combat both metabolic toxicity and infection. In the case of *Candida albicans*, a common yeast that causes opportunistic infections, metformin prevents the fungus from “switching on” crucial virulence functions, including filamentous growth and biofilm formation [61]. *Candida* exists as a round yeast (budding yeast), but can also grow as elongated, branching filaments (hyphae). This shift in morphology is critical for the pathogen to invade tissues, avoid host immune cells, and mature into robust biofilms. Metformin has been found to interfere with several key signaling pathways used by *Candida albicans* to switch to its invasive, filamentous form, and inhibits microbial adherence, matrix assembly, and quorum sensing to prevent biofilm formation [35,62]. Several pathogenic bacterial species form complex communities known as biofilms that cause a wide range of infections in humans, increasing the severity of disease due to increased resistance to the host immune response and to broad-spectrum antimicrobial agents [63,64]. Anti-virulence approaches have attracted increasing interest because they may reduce selective pressures associated with conventional antimicrobial therapy. Experimental studies suggest that metformin may influence selected virulence-associated behaviors and biofilm characteristics; however, the clinical significance of these observations remains uncertain [63,65].

### 3.6. Host-Directed Antimicrobial Activation Through AMPK Signaling

Beyond the experimentally reported effects on microbial physiology, metformin has also been investigated for potential host-directed immunometabolic effects mediated through AMPK signaling pathways [66]. The energy-sensing kinase AMPK is a key regulator of energy homeostasis, also integrating metabolic regulation with immune responses. Metformin-induced activation of AMPK in macrophages enhances autophagic flux and increased phagolysosome fusion resulting in increased killing of microbes [67]. Furthermore, macrophages stimulated with metformin modulate inflammatory signaling to prevent excessive production of pro-inflammatory cytokines while effectively controlling the pathogen, to prevent tissue damage [68].

### 3.7. Synergistic Interaction with Conventional Antimicrobials

Metformin might be of benefit in improving therapy of infections through enhancing activity of existing antimicrobial therapies through synergistic pharmacodynamic interactions. Metformin targets mitochondrial energy metabolism and results in oxidative stress, which is known to compromise fungal biofilms, thereby sensitizing cells to existing antifungal drugs [35]. Furthermore, metformin has been reported to decrease biofilm-mediated resistance mechanisms in bacteria, thereby increasing efficacy of existing antibacterial agents [69,70]. Improved therapy for fungal infections is an emerging need given the increasing number of severe fungal disease cases, including candidiasis, aspergillosis and mucormycosis. Using metformin in combination with standard antifungal therapies such as amphotericin B or caspofungin has shown synergistic activities and enhanced killing of fungal pathogens making this an innovative approach to treating life-threatening infections [71,72]. This multi-domain antimicrobial and osteoimmune effects collectively support the MRIM framework, as summarized in Table 1, which integrates metformin-mediated metabolic reprogramming across microbial, immune, and skeletal compartments through conserved AMPK–mitochondrial–NF-κB signaling pathways.

## 4. Mechanistic Basis of Osteoprotective Effects of Metformin

### 4.1. AMPK Driven Osteogenic Reprogramming

Metformin has been reported to have osteoprotective effects through promotion of osteoblast formation [40,80,83]. The bone marrow contains a pool of MSCs which can differentiate into either osteoblasts or adipocytes. These stem cells respond to a variety of environmental stimulus including nutritional energetic status, pro-inflammatory cytokines, oxidative stress and advanced glycosylation end products. However, in the post-reproductive females, the elderly, or in individuals with chronic infection or conditions of aging, these stem cells are directed toward the adipocyte lineage, resulting in marrow fat accumulation and bone loss [88,89]. Activation of the energy-sensing enzyme AMPK by metformin restores energy-sensing in MSCs, allowing them to be directed back to the osteoblast lineage [79,90].

Activation of AMPK can induce gene expression programs that include transcription factors such as Runt-related transcription factor 2 (RUNX2) and osterix [91]. These transcription factors can drive the expression of genes responsible for forming the osteogenic matrix necessary for mineralization and matrix maturation. This transcriptional program interacts with metabolic regulation during osteoblast differentiation. Metformin can activate AMPK to increase PGC-1α expression leading to an increase in the number of mitochondria required to supply the energy needed for collagen synthesis, alkaline phosphatase, and hydroxyapatite deposition [87,92,93]. In inflammatory bone diseases, such as rheumatoid or osteoporotic bone, mitochondrial dysfunction is a critical component to defective osteogenesis [94].

Infection-associated osteoporosis is an increasingly recognized condition in which AMPK plays a crucial role [95,96]. Infection-induced inflammation is known to inhibit osteoblast differentiation through cytokines TNF-α and IL-1β via NF-κB-mediated gene expression. Metformin stimulates AMPK to reverse the inhibitory effects on osteogenic gene transcription, counteracting the effects of inflammation to enhance the differentiation potential of osteoblasts [97,98]. In this manner, AMPK integrates metabolic, inflammatory and differentiation pathways to modulate the response of osteoblasts within the osteoimmune network.

### 4.2. Mitochondrial Restoration and Redox Homeostasis in Bone

Mitochondrial dysfunction is a central pathological feature in both osteoporosis and infection-driven bone loss. Osteoblasts are highly energy-dependent cells requiring sustained oxidative phosphorylation for matrix synthesis and mineralization. In pathological states characterized by chronic infection or systemic inflammation, mitochondrial respiration is impaired, leading to reduced ATP generation and increased production of ROS. This redox imbalance shifts bone remodeling toward resorption by simultaneously inhibiting osteoblast function and enhancing osteoclast differentiation.

Metformin targets mitochondrial-derived superoxide production by partial inhibition of mitochondrial complex-I [99]. Importantly, this does not result in metabolic compromise of host osteoblasts. Instead, inhibition of complex-I in osteoblasts paradoxically stimulates AMPK-mediated mitochondrial biogenesis and optimizes metabolic function, resulting in re-establishment of redox balance by reducing excessive electron leakage and return of the mitochondrial membrane potential to normal [86,100,101]. Beyond its redox-modulating effects, metformin has also been reported to activate endogenous antioxidant defense pathways, particularly nuclear factor erythroid 2-related factor 2 (NRF2), which may contribute to its protective effects on osteoblasts [102]. Activation of NRF2 promotes the transcription of several antioxidant enzymes, including superoxide dismutase, catalase, and glutathione-related enzymes, thereby enhancing cellular antioxidant capacity. These effects may reduce oxidative stress-induced osteoblast apoptosis and improve the survival and function of osteogenic precursor cells [103]. Importantly, the effects of metformin on reactive oxygen species (ROS) generation appear to be highly context-dependent and compartment-specific. In certain experimental settings, transient increases in mitochondrial ROS may function as adaptive stress signals that enhance antimicrobial or cellular defense responses [104,105]. In contrast, under conditions of chronic inflammation or persistent oxidative injury, metformin may reduce pathological ROS accumulation by improving mitochondrial efficiency, stabilizing electron transport chain activity, and activating endogenous antioxidant pathways [106,107,108,109]. Through these mechanisms, metformin may help preserve extracellular matrix integrity and limit inflammation-induced matrix degradation. This mitochondrial–redox regulatory axis is particularly relevant in infection-associated bone destruction, where pathogen-induced oxidative stress promotes osteoclast activation, enhances bone resorption, and simultaneously suppresses osteoblast-mediated bone formation. Preclinical evidence suggests that metformin may influence this inflammatory–metabolic cycle by modulating mitochondrial function, reducing excessive inflammatory signaling, and improving cellular redox balance, which together may create a microenvironment more favorable for bone repair and skeletal regeneration [102,103,110,111].

### 4.3. Osteoimmune Interface and Inflammatory Recalibration

Bone has now been identified as a hormonally active organ, within which complex interactions occur between immune cells, cytokines, and bone-resident cells. The cell types create a milieu known as the osteoimmune interface, and each of these cell populations is strictly regulated by the others [112]. One of the principal pathologic mechanisms occurring in bone in chronic infection is the creation of a state of disrupted tissue homeostasis through continued innate immune activation. It has been reported that both bacterial and fungal infections promote chronic inflammation in the bone environment through sustained production of potent pro-inflammatory cytokines which further increase the number of osteoclasts. It reduces glycolytic inflammatory polarization and increases oxidative phosphorylation, which mediates repair phenotypes by rerouting macrophage metabolism to an anti-inflammatory phenotype through AMPK activation. As a result, the macrophage transforms from an inflammatory effector cell to a reparative regulatory cell, which prevents osteoclast activation from going above and beyond what is ideal [85,113,114]. Metformin reduces the generation of pro-inflammatory cytokines by inhibiting NF-κB activation and exhibiting anti-inflammatory effects at the cell signaling level [78]. Osteoblasts, other osteoblast lineage cells, and stromal cells that make up the pre-osteoclast precursors produce less RANKL because of metformin’s anti-inflammatory effects on bone metabolism [115]. Metformin increases OPG expression, which lowers the RANKL/OPG ratio and shifts the balance away from bone resorption [83]. For infection-induced bone loss, this inflammatory recalibration of the system is crucial because a microbe’s continuous existence triggers an inflammatory state that produces a highly cytokine-rich environment. This inflammatory positive feedback loop in self-sustaining infection-induced bone loss can be effectively stopped by metformin.

### 4.4. Osteoclast Suppression and Resorptive Control

Osteoclast differentiation is mediated by RANKL signals that activate the transcription of NF-κB, mitogen-activated protein kinase (MAPK) and nuclear factor of activated T cells 1 (NFATc1) [116]. However, in pathological bone destruction such as chronic infection and osteoporosis, these pathways are often hyperactivated leading to excessive bone resorption and destruction of bone tissue. Metformin interferes with osteoclastogenesis at multiple regulatory levels, making it a potent anti-resorptive agent. At the transcriptional level, metformin suppresses NF-κB-mediated gene expression for osteoclastogenesis by preventing the activation of upstream kinases that activate AMPK. As a result, inflammatory transcription factors fail to translocate to the nucleus to upregulate the expression of genes important for osteoclast function, including those that encode the proteolytic enzyme cathepsin K and tartrate-resistant acid phosphatase [32]. At the differentiation level, metformin suppresses RANKL-induced maturation of the osteoclast precursors, and as a result, there is a decrease in the numbers of mature, multinucleated resorptive osteoclasts [81,83]. Importantly, this anti-osteoclastic effect is not merely inhibitory but contextually adaptive. In infection-associated bone loss, osteoclast activity is often dysregulated by inflammatory cytokines and pathogen-derived molecular signals. Metformin restores physiological resorption balance rather than inducing pathological suppression, thereby preserving bone remodeling dynamics essential for skeletal integrity.

### 4.5. Integration of the Infection-Bone Loss Axis

**Figure 1:**
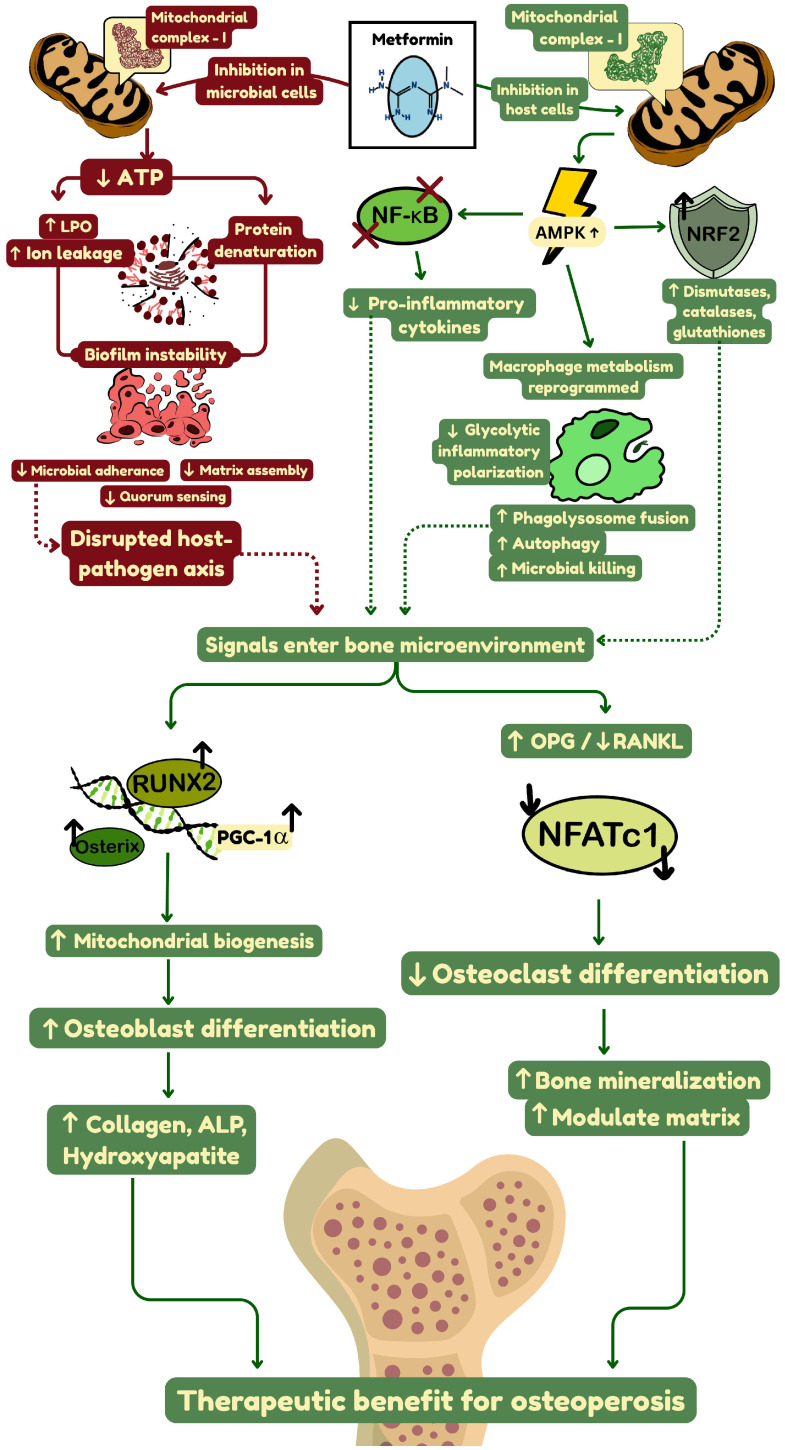
Metformin-mediated immunometabolic regulation within infection-associated bone loss. Abbreviations: AMPK—AMP-activated protein kinase; ATP—Adenosine triphosphate; NF-κB—Nuclear factor kappa B; NRF2—Nuclear factor erythroid 2-related factor 2; PGC-1α—Peroxisome proliferator-activated receptor gamma coactivator 1-alpha; OPG—Osteoprotegerin; RANKL—Receptor activator of nuclear factor kappa-B ligand; NFATc1—Nuclear factor of activated T cells 1; RUNX2—Runt-related transcription factor 2; ALP—Alkaline phosphatase; LPO—Lipid peroxidation.

A major concept emerging from the MRIM framework is that infection-associated bone loss may arise from interconnected immunometabolic and microenvironmental disturbances rather than representing merely a secondary consequence of chronic infection. Persistent biofilm-associated infections caused by organisms such as *Candida albicans* and *Staphylococcus aureus* generate inflammatory and metabolically dysregulated niches characterized by oxidative stress, hypoxia, nutrient competition, mitochondrial dysfunction, and altered bone-cell signaling [117,118,119]. These conditions collectively promote osteoclast activation, impair osteoblast function, and sustain chronic inflammatory responses that contribute to progressive skeletal deterioration. Within this context, metformin is proposed to function primarily as an immunometabolic modulator capable of influencing host inflammatory responses, mitochondrial homeostasis, microbial persistence, and bone remodeling pathways simultaneously. Preclinical evidence suggests that metformin may reduce excessive pro-inflammatory cytokine signaling, improve mitochondrial efficiency, attenuate oxidative stress, and suppress osteoclastogenic pathways while supporting osteoblast survival and differentiation. In parallel, metformin has been reported to interfere with selected biofilm-associated microbial behaviors and persistence mechanisms under specific experimental conditions, although these effects remain context-dependent and incompletely validated clinically. Rather than viewing infection and bone loss as isolated pathological processes, MRIM conceptualizes them as interconnected components of a shared metabolic and inflammatory network involving reciprocal host–pathogen interactions within the bone microenvironment. The integrated mechanistic framework underlying these interactions is summarized in Figure 1, which illustrates the coordinated relationships among mitochondrial regulation, immunometabolic signaling, microbial persistence, inflammatory activation, and skeletal remodeling pathways.

Metformin modulates mitochondrial bioenergetics, inflammatory signaling, host immune responses, and skeletal remodeling pathways implicated in chronic infection-associated bone destruction. In microbial systems, experimental evidence suggests potential effects on biofilm-associated behaviors, oxidative stress adaptation, quorum sensing, and persistence phenotypes. In host cells, metformin-associated AMPK activation has been linked to improved mitochondrial homeostasis, autophagy regulation, restoration of cellular energy balance, and modulation of inflammatory signaling pathways including NF-κB and NRF2. Within bone tissue, metformin has been associated with enhanced osteoblast differentiation and suppression of osteoclastogenic signaling pathways, potentially contributing to preservation of bone integrity under inflammatory conditions. Collectively, these integrated effects support the hypothesis that metformin may influence interconnected host–pathogen–bone metabolic interactions involved in infection-driven bone loss. Solid arrows represent experimentally supported interactions derived from published mechanistic or preclinical evidence, whereas dashed arrows indicate conceptual systems-level relationships intended to stimulate future investigation.

### 4.6. Bone Regeneration and Structural Restoration

Metformin not only lowers inflammation and bone resorption but also actively stimulates bone regeneration by improving osteoblast differentiation and mineralization [81,120]. This action is closely related to its metabolic effects. To establish mineral structures and synthesize collagen, osteoblasts need large amounts of ATP, a process that is hampered by metabolic stress. Increased bone forming capacity will result from mitochondria’s improved energy efficiency and less oxidative damage. Activation of AMPK stimulates the Wnt/β-catenin pathway, which further enhances osteoblast differentiation and proliferation [121,122]. In experimental conditions, this synergy results in better microarchitecture, skeletal integrity, and trabecular bone density. The loss of bone tissue and continuous inflammatory stimulation typically prevent the bone regeneration that occur during chronic infection. By concurrently lowering the inflammatory load and boosting osteogenic signaling, metformin overcomes this inhibition and permits efficient bone regeneration even in damaged microenvironments [40,123].

### 4.7. Skeletal Systems Within MRIM

According to the MRIM framework, immune cells, microbial signals, and stromal components continuously interact to control bone as a dynamic metabolic organ. By focusing on common regulatory components including AMPK, mitochondrial complex I, ROS signaling, and NF-κB pathways, metformin acts as a metabolic reprogramming interface that balances these interactions [38,124]. This integrated view explains why metformin consistently shows benefits for a variety of skeletal conditions, such as inflammatory bone disorders, osteoporosis, and infection-associated bone loss. It is uniquely positioned at the intersection of metabolic pharmacology and osteoimmunology due to its capacity to concurrently control immunological signaling, energy metabolism, and cellular differentiation. Ultimately, MRIM reframes skeletal disease as a systemic metabolic disorder caused by dysregulated host–pathogen–bone interactions rather than as a single structural failure, with metformin serving as a key corrective regulator that can restore multi-layered homeostasis.

## 5. Preclinical and Clinical Evidence Supporting Dual Domain Activity of Metformin

### 5.1. Structure of Translational Evidence

Evidence from in vitro, animal, and human studies suggests that metformin may influence both infection-related processes and skeletal health, supporting investigation of potential mechanistic links between these domains [90,125,126]. When presented using the MRIM, which examines the management of microbial resistance, the underlying infection, and bone loss as signs of metabolic failure, such evidence becomes more convincing. From a research standpoint, metformin has been used to combat a variety of pathogens, such as bacteria and fungi, by directly affecting microbial energy metabolism as well as host immunological pathways, such as boosting the effectiveness of macrophage phagocytosis or triggering autophagosomes formation. Additionally, it improves trabecular architecture, strength, and BMD. Although preliminary, patient data from clinical trials are beginning to reflect this, with studies indicating fewer fractures and occasionally better outcomes in chronic infections in metformin-treated diabetic patients. Collectively, these findings raise the possibility of shared metabolic mechanisms linking infection-related outcomes and skeletal health.

### 5.2. Antimicrobial Translational Convergence

Metformin has also been reported for its antifungal properties [35]. Its mechanism of action may involve some unconventional fungicidal processes which are distinct from the typical mode of action of many antifungal drugs, which are often based on the inhibition of enzymic activity. One of the studies with *Candida albicans* revealed that metformin caused mitochondrial dysfunction, oxidative stress, inhibited accumulation of biofilm biomass and decreased both planktonic and biofilm formation [35]. Fungal biofilms represent a major therapeutic challenge because they exhibit phenotypic tolerance that reduces susceptibility to antimicrobial treatment.

Metformin has been reported to stimulate macrophages to kill bacteria including the major intracellular pathogen *Mycobacterium tuberculosis* [127,128]. The effect occurs through bacterial-induced activation of AMPK in the macrophages to reprogram metabolism to increase autophagy and organelle degradation, enhance phagolysosome acidification and improve mitochondrial function [129]. This host-directed strategy differs from conventional antimicrobial approaches because it primarily targets host cellular responses rather than bacterial resistance mechanisms [130]. Combination of metformin with conventional antimicrobials may suppress fungal stress tolerance pathways, resulting in synthetic suppression and synergy in various experimental systems. In experimental systems, metformin has demonstrated synergistic interactions with established antifungal agents, potentially reducing the concentrations required to achieve antifungal activity [131]. The underlying mechanism of action may translate to clinical applications in the management of drug-resistant fungal infections. The translational relevance of metformin across AMR and osteoporotic bone loss is further supported by convergent preclinical and clinical evidence, as summarized in Table 2, which maps its effects across in vitro, animal, and human studies demonstrating consistent modulation of AMPK-dependent metabolic and inflammatory pathways.

### 5.3. Resistance Reversal Logic and Evolutionary Pressure Modulation

Antimicrobial resistance is a highly complex and evolutionarily dynamic phenomenon involving genetic adaptation, metabolic plasticity, stress-response signaling, and environmental selection pressures. Within the MRIM framework, metformin is not proposed as a direct resistance-reversing antimicrobial agent but rather as a potential metabolic modulator that may influence microbial fitness, virulence-associated behaviors, host immune responses, and biofilm stability under specific experimental conditions. Importantly, most evidence supporting these effects derives from in vitro or preclinical systems, frequently using concentration ranges that may not fully reflect physiological tissue exposure in humans [35,146]. Therefore, any proposed role of metformin in antimicrobial stewardship or resistance modulation should be interpreted cautiously and considered hypothesis-generating rather than clinically established.

A clear distinction should also be made between direct antimicrobial activity and anti-virulence or host-directed immunometabolic effects. In several experimental systems, metformin has been reported to alter microbial behaviors associated with pathogenicity, including biofilm formation, oxidative stress adaptation, quorum sensing, and persistence phenotypes, without necessarily exerting potent bactericidal or fungicidal activity at clinically achievable concentrations [63,64,147,148]. These findings suggest that metformin may influence microbial pathogenic potential or host–microbe interactions under selected metabolic conditions rather than functioning as a conventional antimicrobial agent targeting essential microbial replication pathways. Furthermore, microbial populations possess substantial metabolic adaptability and may activate compensatory survival pathways under metabolic stress conditions [149,150]. Consequently, it would be premature to conclude that metformin imposes universal evolutionary constraints on antimicrobial resistance development. In addition, chronic biofilm-associated infections contain metabolically heterogeneous microbial populations, including dormant persister cells and nutrient-restricted subpopulations, which may variably respond to metabolic interventions. Collectively, these considerations emphasize that the proposed antimicrobial-related effects of metformin remain mechanistically intriguing but require further experimental validation and prospective clinical investigation before definitive translational conclusions can be drawn.

### 5.4. Osteoprotective Translational Continuum

In ovariectomized rodents, a model that mimics postmenopausal osteoporosis due to estrogen deficiency, metformin treatment increases trabecular bone volume and improves cortical thickness and overall bone strength, as demonstrated by both biomechanical testing and micro-computed tomography (µCT) analysis [134,136]. The mechanisms underlying the osteogenic effects of metformin involve AMPK-activated osteoblasts and the suppression of osteoclastogenesis [90,151]. Experimental studies suggest that metformin may influence mesenchymal stem cell differentiation by promoting osteoblastogenesis while reducing adipogenic commitment [79,152,153,154]. These observations raise the possibility that metformin could help preserve osteogenic potential in aging bone marrow, although further validation is required. Importantly, these osteoprotective effects of metformin are not restricted to models of metabolic bone disease. Experimental studies suggest that metformin may attenuate bone loss in selected inflammatory and infection-associated models; however, confirmation in human disease remains limited [103,155].

### 5.5. Osteomyelitis Clinical Bridge Framework

From the perspective of MRIM, chronic osteomyelitis is not viewed as two separate processes—infection and bone destruction—but as a single, tightly linked metabolic condition. Persistent infection and bone loss may interact through reciprocal pathological processes that contribute to disease chronicity. In chronic infections, subsets of bacteria may adopt low-metabolic persister states that reduce susceptibility to antimicrobial therapy and immune clearance [156]. The idea that persister cells are the primary cause of infection persistence and recurrence is becoming more widely acknowledged. As tissue damage, bacterial invasion, and an inflammatory response occur, chronicity gradually develops. In this instance, inflammation persists since it is unable to go away on its own. By activating osteoclasts, this prolonged inflammation promotes bone deterioration while also impairing immune system effectiveness. Under conditions of persistent inflammation and incomplete microbial clearance, persister cells may survive within protected microenvironmental niches, thereby contributing to chronic infection persistence. This leads to the formation of biofilm-associated lesions that are highly resistant to treatment [157,158]. By interfering with the energy metabolism of pathogens like *Staphylococcus aureus*, metformin has been shown to prevent biofilm development [74,159]. By enhancing macrophage function and intracellular bacterial clearance, it also seems to support host immunity. Metformin has also been associated with decreased bone resorption in the setting of bone health by suppressing RANKL-driven osteoclast activity and promoting osteoblast-mediated bone growth [81,126]. Collectively, these observations support the hypothesis that integrated modulation of immune, infectious, and skeletal pathways may represent a useful area for future investigation. In diseases like chronic osteomyelitis, where single-target treatments frequently fail, this type of integrated, multi-level strategy may be particularly pertinent. Metformin stands out as a viable option for additional research in this area due to its long history of clinical usage, good safety profile, oral accessibility, and affordability. Nevertheless, this methodology is still hypothesis-driven, and further targeted translational research will be required to validate its clinical applicability.

### 5.6. Clinical Observational Evidence Synthesis

Observational clinical studies in subjects of all ages with diabetes have shown an association between metformin treatment and a reduced risk of fractures when compared with other diabetes treatments [139,141,160,161]. In addition, studies have shown an improvement in BMD in subjects with diabetes treated with metformin [162,163,164,165]. While studies of this nature are subject to confounders such as the subjects’ glucose control and demographics, large observational studies suggest that metformin has a potential protective effect on the skeleton, which is independent of glucose control. To date, all findings have been obtained from retrospective studies. However, available data suggest that metformin could also be used for the treatment of tuberculosis with reduced mortality and/or faster sputum conversion. Clinical observations suggest that metformin could potentially enhance host immune competence to fight chronic infections. Although there is some indirect evidence to suggest that metformin might have antimicrobial or anti-osteoporotic effects, there are no proper randomized controlled trials (RCTs) performed on this topic. There is a large translational gap which needs to be addressed by future prospective interventional studies assessing both infection and bone outcomes within the same clinical trial.

Available evidence suggests that metformin may influence multiple processes involving metabolism, immune regulation, and tissue remodeling. These effects underpin both the antimicrobial effects of metformin (bioenergetic collapse, oxidative stress and activation of host immunity) and the osteoprotective effects of metformin (activation of osteoblasts via AMPK, inhibition of osteoclasts and redox stabilization). Each domain alone is not sufficient, and they are mechanistically intertwined through shared metabolic pathways. Importantly, microbial survival as well as bone homeostasis depends on mitochondrial function, ROS signaling, autophagy and expression of inflammatory genes, thereby necessitating an integrated view of host skeletal responses. This convergence supports the hypothesis that dysregulated metabolic pathways may represent a common mechanistic link between persistent infection and skeletal degeneration. Within this framework, metformin has attracted increasing interest as a metabolic reprogramming agent, as accumulating evidence suggests that its biological actions extend beyond glucose regulation to the modulation of cellular energetics, inflammatory signaling, and tissue homeostasis.

From the clinical perspective, metformin’s most direct translational uses are found in three areas. First, as an adjunct antimicrobial agent in multidrug-resistant fungal and bacterial infections. Second, as a bone-protective agent in metabolic and inflammatory osteoporosis. Third, as a dual-purpose treatment for bone conditions such as periodontal disease and osteomyelitis that are linked to infections. Future clinical research should concentrate on integrated trial designs that assess bone repair and infection clearance as co-primary outcomes. Such studies would be in line with new systems pharmacology techniques in precision medicine and would mark a substantial shift from traditional single-disease frameworks. These observations support further exploration of metabolic modulation as a potential strategy for addressing complex osteoinflammatory disorders.

### 5.7. Limitations, Translational Challenges, and Future Considerations

Despite increasing interest in metformin as a potential immunometabolic modulator, several important limitations and translational uncertainties must be acknowledged when interpreting the current evidence base. First, much of the mechanistic evidence supporting antimicrobial and osteoprotective effects of metformin derives from in vitro systems, animal studies, or retrospective observational analyses, limiting direct causal inference in human disease. Experimental conditions frequently employ metformin concentrations substantially exceeding clinically achievable plasma or tissue levels, particularly in studies evaluating direct antimicrobial activity against biofilms or metabolically dormant microbial populations. Consequently, whether similar antimicrobial effects occur in vivo within complex human infection microenvironments remains uncertain. Another important limitation relates to pharmacokinetic and tissue penetration challenges. Chronic osteomyelitis, implant-associated infections, and biofilm-mediated diseases are characterized by impaired vascularity, hypoxia, altered extracellular matrix composition, and metabolically heterogeneous microbial communities, all of which may reduce effective drug penetration and influence therapeutic responsiveness. Furthermore, microbial populations exhibit substantial metabolic flexibility and adaptive capacity, enabling survival under nutrient-restricted or oxidative stress conditions. These adaptive mechanisms may limit the long-term effectiveness of metabolic perturbation strategies and complicate assumptions regarding resistance suppression.

Clinical evidence supporting skeletal benefits of metformin also remains heterogeneous. Although several observational studies report associations between metformin exposure and improved bone mineral density or reduced fracture risk, other studies have demonstrated neutral or inconsistent findings. Importantly, many clinical datasets are subject to confounding by glycemic control, body composition, comorbidities, concurrent medications, and patient demographics, making it difficult to determine whether observed skeletal benefits are independent of improved metabolic health. Similarly, evidence supporting adjunctive antimicrobial benefits in chronic infections remains preliminary and largely observational. Potential safety considerations should also be recognized. Metformin use may be limited in patients with severe renal dysfunction, tissue hypoxia, advanced frailty, or critical illness, particularly in settings where altered mitochondrial metabolism could theoretically contribute to metabolic complications. In addition, excessive suppression of inflammatory responses could potentially impair aspects of host defense under certain pathological conditions. These considerations highlight the importance of careful patient stratification in future translational studies. Collectively, these limitations emphasize that MRIM should presently be regarded as a conceptual and hypothesis-generating framework rather than a clinically validated therapeutic paradigm. Future investigations should prioritize physiologically relevant experimental models, concentration-dependent analyses, spatial metabolomics, host–microbe interaction studies, and adequately powered prospective clinical trials evaluating both infectious and skeletal outcomes simultaneously. Such studies will be essential to determine whether metabolic modulation can provide reproducible therapeutic benefit across complex osteoinflammatory disease states.

## 6. Metabolic Reprogramming Interface Model

### 6.1. Conceptual Overview and Systems-Level Architecture of MRIM

The Metabolic Reprogramming Interface Model (MRIM) is proposed as a multiscale conceptual framework in which metabolic processes function as a dynamic interface linking host immunity, microbial adaptation, and skeletal remodeling. Rather than focusing on a single pathway, MRIM proposes that disease progression emerges from interactions among interconnected metabolic networks across molecular, cellular, and tissue levels. Within this framework, cellular metabolism functions not only in energy production but also in regulating signaling pathways, gene expression, and cellular phenotypes.

Within the immune compartment, cellular metabolism is tightly coupled with functional polarization. Pro-inflammatory macrophages predominantly utilize aerobic glycolysis to support rapid ATP generation and inflammatory mediator production, whereas anti-inflammatory or reparative macrophages rely more heavily on oxidative phosphorylation and mitochondrial integrity [166,167,168,169]. Accordingly, perturbations in energy metabolism may influence immune polarization, although the direction and magnitude of these effects remain highly context-dependent, shaped by environmental cues and substrate availability.

Microbial populations, particularly those organized in biofilms, exhibit substantial metabolic plasticity that supports survival under nutrient limitation, hypoxia, and immune pressure. These adaptations include metabolic downregulation, utilization of alternative substrates, and activation of stress-response pathways that collectively enhance persistence. Importantly, while host-directed metabolic perturbations may constrain microbial fitness, pathogens also possess significant adaptive capacity, and therefore metabolic vulnerability must be interpreted within the context of microbial resilience and evolutionary dynamics.

At the skeletal level, metabolic regulation is central to bone homeostasis. Osteoblasts depend primarily on mitochondrial oxidative phosphorylation to support matrix synthesis and mineralization, whereas osteoclasts rely more on glycolysis during active resorptive phases [170,171,172]. These distinct metabolic requirements suggest that metabolic interventions may differentially affect bone cell populations, thereby altering remodeling balance in a context-dependent manner. Bone marrow adipocytes, which also exhibit a predominantly oxidative metabolic profile, may similarly be affected, with implications for mesenchymal lineage allocation and adipogenic differentiation.

Within MRIM, these metabolic differences are integrated into a unified system in which metabolic competition, signaling crosstalk, and environmental constraints collectively shape disease trajectories, particularly in conditions where infection, inflammation, and bone loss coexist. In this context, metformin-induced inhibition of mitochondrial oxidative phosphorylation is expected to differentially impact cell populations based on their metabolic dependencies. Osteoblasts and adipocytes, which rely heavily on mitochondrial respiration, may experience reduced biosynthetic and functional capacity, whereas osteoclasts, with greater glycolytic reliance during resorptive activity, may exhibit altered sensitivity to mitochondrial perturbation. This divergence provides a mechanistic basis for context-dependent modulation of bone remodeling under metformin exposure.

To ensure rigorous interpretation and avoid conflation of established findings with conceptual synthesis, MRIM incorporates a structured stratification of evidence across three levels. The first level includes experimentally validated mechanisms, such as metformin-induced activation of AMPK via mitochondrial modulation, along with its established effects on cellular energy balance and inflammatory signaling pathways, including NF-κB. These form the foundational experimental basis of the framework. The second level comprises mechanistically supported but context-dependent interactions, including reported effects of metformin on macrophage polarization, osteoclast differentiation, osteoblast activity, and microbial behavior in experimental systems. These findings, while supported by preclinical data, exhibit variability across models and may not directly translate to complex in vivo conditions. The third level includes higher-order theoretical constructs, such as coordinated host–pathogen metabolic competition and the proposed links between metabolic dysregulation, antimicrobial persistence, and bone loss. These represent hypothesis-generating concepts intended to guide future experimental validation rather than definitive mechanistic conclusions.

### 6.2. Metformin as a Multilevel Immunometabolic Regulator Within MRIM

Within the MRIM framework, metformin is conceptualized as a mitochondrial bioenergetic regulator that exerts its primary action through partial inhibition of complex I of the electron transport chain. This leads to reduced OXPHOS, decreased ATP production, and increased AMP levels, thereby activating AMPK. A key downstream consequence is a metabolic shift from OXPHOS toward glycolysis, which is considered the primary upstream driver of metformin’s pleiotropic biological effects. Accordingly, downstream outcomes such as modulation of inflammatory signaling, immune cell function, and cellular differentiation are interpreted as secondary consequences of altered cellular energetics.

This metabolic reprogramming produces cell type-specific effects depending on bioenergetic dependency. OXPHOS-dependent cells such as osteoblasts and adipocytes may exhibit reduced biosynthetic capacity, whereas glycolysis-prone cells, including activated macrophages and osteoclasts, undergo functional reprogramming rather than uniform suppression. Thus, metformin functions as a modulator of metabolic flux distribution across heterogeneous cellular populations rather than a uniform inhibitor or activator.

At the systems level, MRIM positions metformin as an immunometabolic regulator acting at the interface of host–pathogen interactions and skeletal microenvironment dynamics. Disease progression is conceptualized as a network disturbance involving mitochondrial dysfunction, metabolic reprogramming, inflammatory signaling, microbial persistence, and impaired tissue repair. Within this framework, metformin is proposed to influence conserved regulatory nodes including AMPK signaling, redox balance, autophagy, mitochondrial function, and NF-κB-mediated inflammatory pathways. These integrated effects collectively shape microbial survival strategies, host immune responses, and skeletal remodeling processes under chronic inflammatory conditions.

In chronic infection-associated bone disease, MRIM further proposes a metabolically competitive microenvironment in which pathogens, immune cells, and skeletal cells compete for nutrients and oxygen. Persistent pathogens may exploit metabolic dysregulation to establish biofilm-associated survival niches, while host tissues activate inflammatory and reparative responses to restore homeostasis [173,174,175]. Within this context, metformin is viewed not as a direct antimicrobial or bone-specific agent, but as a context-dependent metabolic modulator capable of influencing multiple interconnected pathological processes.

### 6.3. Causal Loop Dynamics of the Infection–Bone Metabolic Axis

A critical yet often underemphasized determinant of the infection–bone metabolic axis is the intrinsic metabolic adaptability of microbial pathogens. Bacteria can dynamically reprogram their metabolic pathways in response to environmental stressors such as oxygen limitation, nutrient deprivation, and host-imposed metabolic constraints. These adaptations include shifts between oxidative respiration and fermentative metabolism, utilization of alternative carbon sources, and transition into low-metabolic states associated with biofilm formation and persistence. Collectively, these strategies enable microbial survival within metabolically heterogeneous and immunologically hostile host microenvironments.

Within this adaptive landscape, metformin is unlikely to exert its effects through direct antimicrobial activity alone. Instead, MRIM proposes that metformin indirectly influences microbial viability by reshaping host systemic and local metabolic conditions, including reduced circulating glucose levels, altered lactate dynamics, and changes in tissue oxygen utilization. These metabolic shifts modify the nutrient landscape and may constrain microbial growth or promote metabolic state transitions. Accordingly, antimicrobial effects are interpreted within MRIM as emergent outcomes of host–pathogen metabolic competition rather than direct pharmacological targeting.

From a translational standpoint, it is essential to distinguish conventional antibiotic mechanisms from host-mediated metabolic modulation. Metformin does not function as a direct antimicrobial agent; rather, its systemic effects on glucose homeostasis, cellular energetics, and immune regulation collectively create an environment that may be less permissive for microbial proliferation. This distinction is critical for accurate interpretation of experimental findings and for positioning metformin as an adjunct metabolic modulator rather than a primary antimicrobial therapy.

Chronic infection-driven inflammation constitutes the central reinforcing loop within the MRIM pathogenic network. Persistent microbial presence sustains activation of innate immune pathways, resulting in continuous production of pro-inflammatory cytokines including TNF-α, IL-1β, and IL-6 [176]. These mediators disrupt bone homeostasis by enhancing RANKL signaling, promoting osteoclast activation, and suppressing osteoblast function, thereby shifting the balance toward net bone resorption [177]. This establishes a self-perpetuating cycle in which inflammation drives bone loss, which in turn further destabilizes tissue integrity [19].

Simultaneously, microbial biofilm formation reinforces this loop by enhancing pathogen persistence. Within biofilms, bacteria exhibit reduced metabolic activity, increased stress resistance, and enhanced immune evasion, allowing long-term survival despite ongoing host immune pressure. This persistence maintains chronic inflammatory signaling, thereby prolonging osteolytic activity and tissue damage [113,178].

Within this context, MRIM conceptualizes metformin as a multi-nodal disruptor of the infection–inflammation–bone feedback loop rather than a single-pathway agent. On the microbial side, metformin-induced energetic stress may reduce biofilm resilience and limit pathogen persistence. At the immune level, AMPK activation promotes a shift in macrophage polarization toward a less pro-inflammatory phenotype, thereby reducing cytokine production [32,85]. In bone tissue, metformin-mediated inhibition of NF-κB signaling is proposed to suppress osteoclast-driven resorption while supporting osteoblast activity [81,179,180]. Collectively, these interconnected effects are hypothesized to shift the system away from a self-sustaining destructive state toward a more regulated environment conducive to tissue repair and homeostasis.

### 6.4. Hierarchical Translational Organization

**Figure 2:**
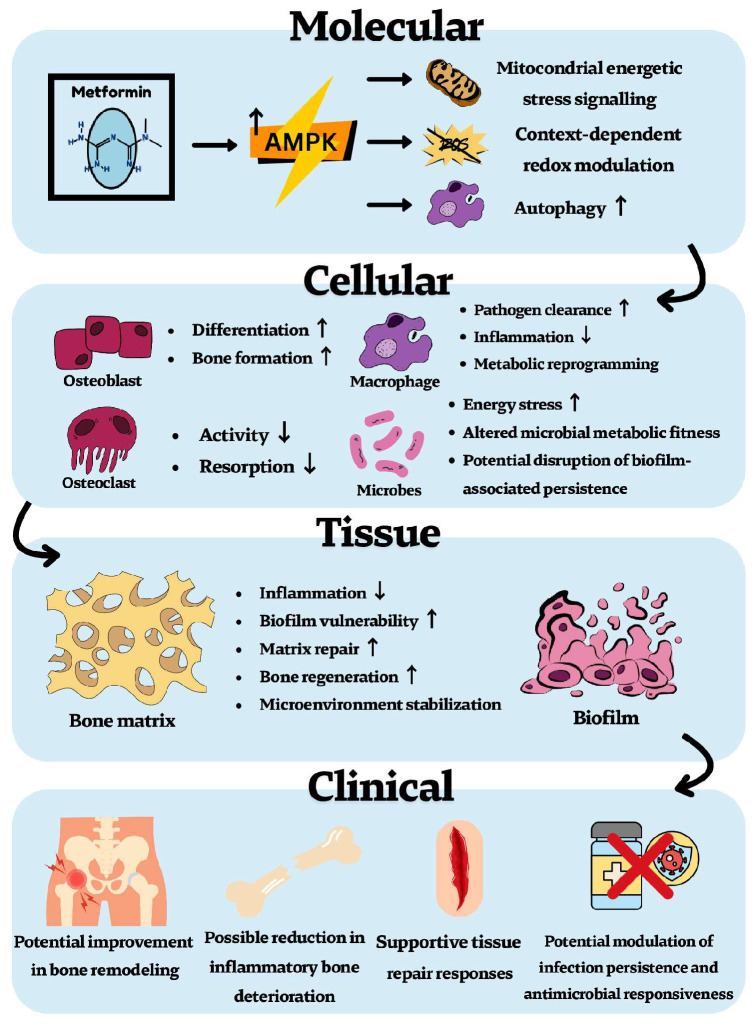
Hierarchical translational effects of metformin across molecular cellular tissue and clinical levels. Abbreviations: AMPK—AMP-activated protein kinase; ROS—Reactive oxygen species.

The evolution of metformin’s influence across biological scales, from the molecule to the entire organism, could help visualize the MRIM framework. Metformin interacts at the cellular level in the first layer, affecting mitochondrial metabolic systems and altering ATP and AMP levels as well as energy generation. Numerous cascade signals, including AMPK activation, changes in ROS levels, and modifications to autophagy pathways, will follow these initial changes [32,179,181]. Cell’s functional traits change as these signals spread across the cells. Microbes experience increased energy stress, which reduces their growth, weakens biofilm formation, and lowers their resistance to oxidative stress [182]. At the same time, immune cells such as macrophages become more efficient at clearing pathogens while generating fewer excessive inflammatory responses [183]. Stem cells are directed toward osteoblast differentiation, whereas osteoclast activity decreases, leading to reduced bone resorption. At the tissue level, these coordinated effects result in reduced inflammation, improved blood flow stability, and progressive repair of the extracellular matrix. Biofilms become more vulnerable and easier to eliminate, while new bone formation is reactivated, supporting overall healing and regeneration. Collectively, these molecular and cellular changes translate into clinical outcomes, including reduced infection burden, increased bone density, lower fracture risk, and improved patient response to therapy. The hierarchical, multiscale organization of these effects is illustrated in Figure 2, which depicts the translational continuum of metformin action from molecular signaling events to cellular reprogramming, tissue-level remodeling, and ultimately clinical outcomes.

This figure illustrates the multiscale MRIM framework of metformin action from molecular to clinical levels. At the molecular level, metformin activates AMPK through mitochondrial modulation, leading to altered ATP/AMP balance, reduced ROS, and enhanced autophagy. At the cellular level, these effects induce metabolic stress in microbes (reducing growth and biofilm formation), enhance macrophage-mediated pathogen clearance while limiting inflammation, promote osteoblast differentiation, and suppress osteoclast activity. At the tissue level, coordinated responses result in reduced inflammation, increased biofilm susceptibility, improved extracellular matrix repair, and enhanced bone regeneration. These integrated biological effects may contribute to clinically relevant outcomes such as preservation of bone mineral density, improved tissue repair, and altered susceptibility to infection; however, many of these relationships remain incompletely validated in prospective clinical studies.

### 6.5. Microbial Host–Bone Metabolic Competition Model

One hypothesis within the MRIM framework is that host cells and pathogens may compete for limited metabolic resources within inflammatory microenvironments, thereby influencing immune responses, microbial persistence, and tissue remodeling. Microbes frequently acquire the upper hand in chronic infections by changing their metabolism, which enables them to endure conditions with low oxygen or food levels. Because of this, they have an edge over immune cells, particularly in small areas like diseased bone tissue [184,185]. Experimental observations suggest that metformin may influence certain aspects of host and microbial metabolism under selected conditions. However, the extent to which these effects alter host–pathogen metabolic interactions in vivo remain uncertain and requires further investigation.

### 6.6. Predictive Clinical Framework and Stratified Response Modeling

An important translational implication of the MRIM framework is the possibility that metabolic and inflammatory heterogeneity may influence therapeutic responsiveness to immunometabolic modulation. The model proposes that patients with chronic inflammatory states, metabolic dysregulation, biofilm-associated infections, or combined osteoinflammatory disease may exhibit distinct metabolic microenvironment profiles that could potentially influence responsiveness to adjunctive metformin therapy. Metformin-associated effects may be more pronounced in disease settings characterized by heightened inflammatory signaling, mitochondrial dysfunction, impaired metabolic homeostasis, or persistent infection-associated tissue remodeling. However, these concepts remain preliminary and require prospective validation. Importantly, the framework does not imply uniform clinical responsiveness across all osteoporotic or infectious conditions, and therapeutic effects are likely to vary according to metabolic status, microbial composition, inflammatory burden, tissue microenvironment, and host comorbidities. Consequently, MRIM supports the future development of stratified or precision-based approaches integrating metabolic, inflammatory, skeletal, and infectious biomarkers to identify patient subgroups that may potentially benefit from immunometabolic modulation strategies. Nevertheless, these predictive concepts remain hypothesis-generating and should not yet be interpreted as clinically validated therapeutic algorithms.

### 6.7. Osteomyelitis Systems Collapse and Repair Model

Within the proposed MRIM framework, osteomyelitis may be conceptualized as a condition in which persistent infection, inflammation, and bone remodeling abnormalities interact through interconnected biological processes. Pathogens can thrive in protected areas created by biofilms that grow within bone, protected from both immune system attack and antimicrobial medications. The immune system is continuously activated by these persistent infections, yet the microbes are not effectively eliminated. Osteoclasts are activated by this persistent inflammation, which also suppresses the cells that produce new bone. Over time, there is a significant shift in the balance toward bone loss, which results in structural weakness and poor healing. In this perspective, osteomyelitis is a dynamic cycle in which bone loss, inflammation, and infection all promote one another rather than being a straightforward infection. Theoretically, metformin could function as a metabolic modulator capable of influencing several pathways implicated in infection-associated bone remodeling; however, this concept remains speculative and has not been clinically validated. Metformin impairs microbial survival by reducing microbial energy production, while simultaneously restoring osteogenic activity and enhancing the efficiency of host immune responses. It has been hypothesized that coordinated modulation of metabolic, inflammatory, and skeletal pathways may help disrupt processes contributing to disease persistence, although this proposition requires experimental confirmation.

### 6.8. Model Validation and Falsifiability Framework

The ability to assess and challenge MRIM using experimental data is a useful feature. According to the model, metformin should result in coordinated changes in several important biological markers, such as AMPK activity, mitochondrial function, ROS levels, and the ratio of OPG to RANKL. The validity of the model would be questioned if these changes did not happen simultaneously. Similarly, the model predicts that the antibacterial and bone-protective properties of metformin should be diminished if AMPK signaling is suppressed either pharmacologically or genetically. This offers a straightforward method of determining whether AMPK has a major role. Examining whether biofilms continue to be metabolically active despite metformin treatment would be another test that might reveal different resistance mechanisms not currently accounted for. In general, MRIM presents metformin as a regulator of interrelated biological systems rather than a medication working on a single pathway. It offers a structured approach to comprehending and possibly treating complicated illnesses where infection, immunity, and tissue remodeling are closely linked by connecting molecular events to clinical consequences and by providing specific sites for experimental confirmation.

## 7. Conclusions

The Metabolic Reprogramming Interface Model (MRIM) proposed in this review serves as a systems-level, hypothesis-generating framework that integrates immunometabolism, host–pathogen interactions, and skeletal remodeling within a shared metabolic landscape. Rather than considering antimicrobial resistance, chronic infection, inflammation, and osteoporotic bone loss as isolated pathological entities, MRIM conceptualizes these processes as interconnected biological phenomena that may converge through dysregulated cellular energetics, mitochondrial dysfunction, redox imbalance, and inflammatory signaling. By bringing together evidence from multiple disciplines, the framework offers a structured perspective for exploring how metabolic reprogramming may influence the dynamic interplay between infection, immune responses, antimicrobial tolerance, and skeletal degeneration.

Within this framework, metformin is discussed primarily as a metabolic modulator capable of influencing several conserved regulatory pathways, including AMPK signaling, mitochondrial bioenergetics, oxidative stress responses, autophagy, and inflammatory transcriptional networks such as NF-κB. Emerging preclinical evidence suggests that modulation of these pathways may affect microbial persistence, immune cell polarization, osteoblast differentiation, osteoclast activity, and bone remodeling dynamics. Rather than targeting a single pathological process, metformin appears to exert pleiotropic effects through interconnected mechanisms that may simultaneously influence host defense, inflammatory regulation, and skeletal homeostasis.

However, the biological effects of metformin remain highly context-dependent and are influenced by cell type, metabolic status, disease microenvironment, and experimental conditions. Importantly, many of the mechanistic relationships incorporated within MRIM remain inferential and are derived predominantly from in vitro studies, animal models, mechanistic observations, and retrospective clinical associations rather than definitive causal clinical evidence. Several translational uncertainties therefore remain. Direct antimicrobial activity of metformin at clinically achievable concentrations has not been conclusively established, particularly in complex biofilm-associated infections and poorly vascularized bone environments. Similarly, although observational studies have reported associations between metformin exposure and reduced fracture risk or improved infection-related outcomes, these findings may be influenced by confounding factors such as glycemic control, comorbidities, treatment selection bias, and patient heterogeneity. Furthermore, microbial metabolic plasticity, adaptive resistance mechanisms, and tissue-specific pharmacokinetic constraints may substantially affect therapeutic responses in vivo. Consequently, the concept of metformin as a resistance-modifying or dual antimicrobial–osteoprotective therapeutic strategy should be interpreted with appropriate caution.

A major contribution of MRIM is its emphasis on metabolic regulation as a shared biological interface linking immune function, microbial adaptation, and skeletal homeostasis. By clearly distinguishing experimentally validated mechanisms from context-dependent observations and higher-order theoretical integrations, the framework provides a structured approach for evaluating complex cross-disciplinary evidence while maintaining scientific rigor. This distinction is critical for minimizing overinterpretation of associative findings and supporting the development of reproducible translational research.

Importantly, MRIM should be regarded as a conceptual framework rather than a validated biological model. Future studies should prioritize mechanistic validation using spatially resolved, cell-specific, and longitudinal experimental systems capable of recapitulating the complexity of chronic infection-associated bone disease. In parallel, prospective clinical investigations incorporating both infectious and skeletal endpoints will be required to determine whether metabolic modulation can be translated into clinically meaningful therapeutic strategies. Until such evidence becomes available, MRIM should be viewed as a platform for interdisciplinary investigation and hypothesis generation. Nevertheless, it provides a potentially valuable framework for understanding how metabolic reprogramming may contribute to the interconnected processes of infection, inflammation, antimicrobial tolerance, and skeletal deterioration, thereby identifying new avenues for future research and therapeutic innovation.

## Figures and Tables

**Table 1:** Metformin-mediated metabolic reprogramming across host–pathogen–bone interface within the MRIM framework.

Biological Domain	Cellular System	Metformin Target Node	Functional Outcome	MRIM Interpretation
Microbial system	*Candida albicans*, *Staphylococcus aureus*, *Pseudomonas aeruginosa* biofilms [35,63,73,74]	Mitochondrial Complex I inhibition	ATP depletion, ROS overload, biofilm collapse	Energetic failure of pathogen survival network
Microbial system	Intracellular pathogens [51,75]	Autophagy disruption	Loss of persistence niche	Breakdown of intracellular survival strategies
Immune system	Macrophages [76]	AMPK activation	Enhanced phagolysosomal activity	Immune metabolic reprogramming
Immune system	Cytokine network [77,78]	NF-κB suppression	Reduced TNF-α, IL-6, IL-1β	Anti-inflammatory recalibration
Bone system	Osteoblast lineage [38,79,80]	AMPK–RUNX2 activation	Enhanced osteogenesis	Restoration of bone formation
Bone system	Osteoclast precursors [81,82,83,84]	RANKL inhibition	Reduced osteoclast differentiation	Anti-resorptive shift
Integrated system	Bone–infection niche [85,86,87]	ROS–mitochondrial axis	Redox stabilization	Collapse of infection–bone degradation loop

**Table 2:** Translational evidence mapping of metformin in antimicrobial resistance and osteoporotic bone loss.

Evidence Level	Disease Model	Metformin Effect	Translational Relevance	MRIM Domain
In vitro	*Candida albicans* biofilm [35,132]	Biofilm disruption, mitochondrial collapse	Antifungal adjuvant potential	Microbial metabolism
In vitro	Macrophage infection model [75,133]	Enhanced autophagy, pathogen clearance	Host-directed therapy	Immune metabolism
Animal model	Ovariectomized rodents [134,135,136]	Increased BMD, trabecular preservation	Osteoporosis reversal	Bone remodeling
Animal model	Osteomyelitis model [40,137]	Reduced infection + bone loss	Dual therapeutic effect	Integrated MRIM axis
Clinical cohort	Type 2 diabetes patients [138,139,140,141,142]	Reduced fracture incidence	Osteoprotective association	Skeletal domain
Clinical cohort	Tuberculosis patients [128,143,144]	Improved treatment response	Adjunct antimicrobial effect	Infection control
Integrated synthesis	Multi-system analysis [32,38,124,145]	AMPK–ROS–NF-κB convergence	Unified metabolic therapy model	Whole MRIM network

## Data Availability

All data arising from this study are included within the article.

## Abbreviations

The following abbreviations are used in this manuscript:

AMR	Antimicrobial resistance
AMPK	AMP-activated protein kinase
AMP	Adenosine monophosphate
ATP	Adenosine triphosphate
BMD	Bone mineral density
IL-1β	Interleukin-1 beta
IL-6	Interleukin-6
MAPK	Mitogen-activated protein kinase
MRIM	Metabolic Reprogramming Interface Model
MSCs	Mesenchymal stem cells
μCT	Micro-computed tomography
OXPHOS	Oxidative phosphorylation
NF-κB	Nuclear factor kappa-B
NFATc1	Nuclear factor of activated T cells 1
NRF2	Nuclear factor erythroid 2-related factor 2
ODRI	Orthopedic device-related infection
OPG	Osteoprotegerin
PGC-1α	Peroxisome proliferator-activated receptor gamma coactivator 1-alpha
RANKL	Receptor activator of nuclear factor kappa-B ligand
RCTs	Randomized controlled trials
ROS	Reactive oxygen species
RUNX2	Runt-related transcription factor 2
TNF-α	Tumor necrosis factor alpha
ALP	Alkaline phosphatase
Wnt/β-catenin	Wingless-related integration site/beta-catenin
